# Synthesis and Properties of Fluorinated Hydrophobically Associating Polymers

**DOI:** 10.3390/ma19122599

**Published:** 2026-06-17

**Authors:** Zhonghong Liang, Chungui Li, Yong Qi

**Affiliations:** 1 No. 2 Gas Recovery Plant of Sinopec Southwest Oil & Gas Company, Langzhong 637450, China; liangzhonghong.xnyq@sinopec.com; 2 Western Branch of Jidong Oilfield, China National Petroleum Corporation (CNPC), Yulin 719000, China; lichungui@petrochina.com.cn; 3 College of Chemical Engineering, Sichuan University of Science & Engineering, Zigong 643000, China

**Keywords:** fluorinated hydrophobically associating polymer, aqueous solution polymerization, salt tolerance, shear resistance, enhanced oil recovery

## Abstract

To enhance the temperature, salt and shear resistance of conventional polyacrylamide oil-displacing agents, a fluorinated hydrophobically associating polymer AM-AA-HDFDMA was synthesized by aqueous solution polymerization. Acrylic acid (AA) and acrylamide (AM) were utilized as comonomers, with perfluorooctylethyl methacrylate (HDFDMA) serving as the fluorinated hydrophobic functional monomer. The synthesis employed an ammonium persulfate-sodium bisulfite (APS-NaHSO_3_) redox initiation system. The selected synthesis conditions were established as a total monomer mass fraction of 25%, an initiator dosage of 0.3 wt% of the total monomers, a reaction temperature of 40 °C, and a reaction time of 4.5 h. The chemical structure and micromorphology of the polymer were characterized by Fourier transform infrared spectroscopy (FT-IR), proton nuclear magnetic resonance (^1^H-NMR), and scanning electron microscopy (SEM). Furthermore, its thickening performance, shear resistance, temperature resistance, salt tolerance, and oil displacement efficiency were systematically evaluated.

## 1. Introduction

With growing global energy demand and gradual maturity of conventional oilfields, enhanced oil recovery (EOR) technologies become essential for stable crude oil production [1,2,3]. Among these, polymer flooding has emerged as one of the most widely implemented chemical flooding techniques, owing to its capability to effectively increase the viscosity of displacement fluids, reduce the water–oil mobility ratio, and expand the sweep efficiency [4,5,6]. Currently, partially hydrolyzed polyacrylamide (HPAM) is the predominant polymer utilized for industrial oil displacement [7,8]. However, as exploration and development shift toward harsh reservoir environments characterized by high-temperature and high-salinity (HTHS) conditions, traditional HPAM exhibits significant performance deficiencies. In formation water containing high concentrations of metal cations—particularly divalent ions such as Ca^2+^ and Mg^2+^ [9,10]—electrostatic shielding effects drastically weaken the electrostatic repulsion between the carboxyl groups on the HPAM molecular chains. This tends to make polymer chains coil from an extended conformation, macroscopically leading to an obvious viscosity drop, namely the salt-sensitive effect [11,12]. Furthermore, high shear forces encountered during high-speed flow through wellbores and porous media lead to irreversible mechanical degradation of the HPAM backbone [13,14,15]. Consequently, the development of novel polymers with exceptional thermal stability, salt tolerance, and shear resistance remains a critical scientific and engineering challenge in the EOR field.

To overcome the inherent deficiencies of HPAM, hydrophobically associating water-soluble polymers (HAWSPs) have garnered significant research interest [16,17,18]. By incorporating a small fraction of hydrophobic groups—such as long-chain alkyl or aryl groups—onto the hydrophilic polymer backbone, HAWSPs can self-assemble in aqueous solutions via hydrophobic interactions, forming dynamic intra and intermolecular physical cross-linking networks [19,20]. This reversible three-dimensional network endows the polymers with characteristic shear-thinning and shear-recovery properties [21]. Furthermore, at certain salt concentrations, the intensified polarity of the aqueous environment can actually promote hydrophobic association, leading to a phenomenon known as the “anti-polyelectrolyte effect” [22,23]. However, conventional hydrocarbon-based hydrophobic monomers exhibit distinct limitations. On one hand, excessively long alkyl chains drastically reduce the water solubility of the polymer, failing to meet the requirements for rapid dissolution during field applications [24,25]. On the other hand, under extremely high temperatures, the intensified thermal motion of the hydrocarbon chains tends to disrupt the fragile physical cross-linking points, resulting in the eventual disintegration of the network [26]. Consequently, there is an urgent need to identify novel functional monomers characterized by stronger associative capabilities and superior stability.

In recent years, fluorinated polymers have garnered significant attention in the field of materials science due to their unique physicochemical properties [27,28]. Compared to the C-H bond (413 kJ/mol), the C-F bond (485 kJ/mol) possesses higher bond energy and exceptionally low polarizability, which endows fluorinated functional groups with superior thermal stability and chemical inertness [29,30]. More critically, the strong electronegativity of fluorine atoms confers fluorocarbon chains with both intense hydrophobic and oleophobic characteristics (manifested as extremely low surface free energy). Within aqueous systems, fluorocarbon groups exhibit a much stronger solvophobic effect compared to hydrocarbon counterparts of equivalent chain length [31,32,33]. Consequently, incorporating a minimal molar fraction of fluorinated monomers into the polymer backbone can trigger robust intermolecular association at a lower critical association concentration (CAC), facilitating the construction of a dense and resilient three-dimensional spatial network [34,35]. Furthermore, the rigid, highly hydrophobic/oleophobic structures of the fluorocarbon microdomains effectively withstand thermal fluctuations induced by high temperatures and resist the intrusion of high-salinity ions [36,37]. However, systematic investigations into the rheological behavior of fluorinated hydrophobically associating polymers in complex oilfield fluid systems, as well as their microscopic oil displacement mechanisms within porous media, remain relatively limited.

However, existing fluorinated HAPs either face the contradiction between fluorocarbon chain length, association strength and water solubility, or require complex synthesis processes difficult to scale up. Meanwhile, systematic mechanism research and reservoir adaptability evaluation under coupled harsh conditions are still insufficient. Based on the above theoretical basis, a novel fluorinated hydrophobically associating polymer AM-AA-HDFDMA was synthesized via aqueous free radical copolymerization of acrylamide (AM), acrylic acid (AA) and fluorinated monomer HDFDMA. The molecular structures and three-dimensional micromorphology of the synthesized polymers were meticulously characterized using Fourier-transform infrared spectroscopy (FT-IR), proton nuclear magnetic resonance (^1^H-NMR), and scanning electron microscopy (SEM). Furthermore, a systematic comparative study was conducted to investigate the rheological response characteristics of the novel polymer versus the traditional binary AM-AA copolymer under varying conditions of concentration, temperature, high salinity (including Na^+^, Ca^2+^, and Mg^2+^), and intense mechanical shear. The evolution mechanism of the associative network triggered by fluorinated groups was also explored in depth. Finally, the enhanced oil recovery (EOR) potential of the fluorinated polymer during high water-cut stages was quantitatively evaluated through laboratory sand-pack physical simulation experiments. This research aims to elucidate the physicochemical mechanisms by which fluorinated hydrophobic associations reinforce the polymer chain topology, thereby providing a promising novel chemical agent and a solid theoretical framework for deep profile control and polymer flooding in complex and harsh reservoir environments.

The core innovations of this work, which fundamentally distinguish our system from existing fluorinated/conventional HAPs, are as follows: (1) We adopt simple aqueous solution polymerization to synthesize long fluorocarbon chain polymer AM-AA-HDFDMA, balancing strong association ability and water solubility, avoiding the complex synthesis process of existing systems; (2) We clarify the stabilization mechanism of fluorinated dynamic network under harsh reservoir conditions; (3) The polymer achieves ultra-low CAC and excellent EOR efficiency, with great industrial application potential.

## 2. Experimental Section

### 2.1. Materials

Calcium chloride (CaCl_2_), magnesium chloride (MgCl_2_), sodium chloride (NaCl), sodium sulfate (Na_2_SO_4_), potassium chloride (KCl), sodium bicarbonate (NaHCO_3_), acrylamide (AM), acrylic acid (AA), anhydrous ethanol, sodium bisulfite (NaHSO_3_), ammonium persulfate (APS), and octylphenol polyoxyethylene ether (OP-10) were purchased from Chengdu Kelong Chemicals Co., Ltd. (Chengdu, China). Perfluorooctylethyl methacrylate (HDFDMA) was obtained from Shanghai Aladdin Bio-Chem Technology Co., Ltd. (Shanghai, China). All chemicals were of analytical reagent (AR) grade and used as received without further purification. The ionic compositions of the injection water and formation water are summarized in Table 1.

### 2.2. Methodology

#### 2.2.1. Synthesis of Hydrophobically Associating Salt-Tolerant Polymer

**Figure 1:**
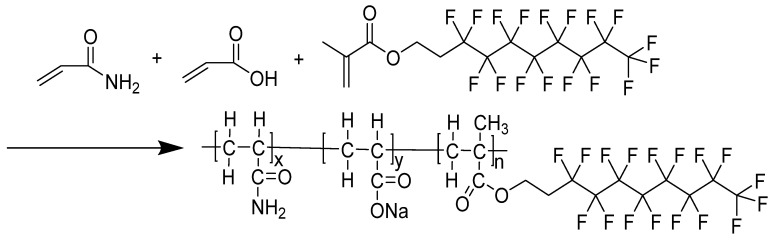
Synthesis scheme of AM-AA-HDFDMA.

A total of 6.25 g of AA was dissolved in 25 g of deionized water in a beaker. The solution pH was adjusted to 7.0 using a sodium hydroxide (NaOH) solution to neutralize the AA into its sodium salt, thereby providing a stable and suitable neutral reaction environment. According to the pKa value of acrylic acid (4.26), the neutralization degree of AA was calculated to be over 99% at pH 7.0, indicating that almost all acrylic acid was converted into sodium acrylate. Subsequently, 6.25 g of AM, OP-10, and HDFDMA (0.10 g) were added sequentially, with the mass ratio of OP-10 to HDFDMA maintained at 1:1. Deionized water was then added to ensure a total monomer mass fraction of 25% relative to the total reaction solution. The resulting mixture was transferred to a blue-capped reagent bottle, sealed with plastic wrap and a screw cap, and subjected to ultrasonic treatment for 30 s to ensure the uniform dispersion of the fluorinated hydrophobic monomers. The bottle was then placed in a water bath at room temperature and purged with nitrogen for 5 min to eliminate dissolved oxygen. A composite initiator system (APS and NaHSO_3_ in a 1:1 molar ratio) was introduced at a dosage of 0.3 wt% relative to the total monomer mass. After thorough stirring, the bottle was sealed and transferred to a constant temperature water bath at 40 °C for a reaction period of 4.5 h to obtain the fluorinated hydrophobically associating polymer, AM-AA-HDFDMA. The crude polymer was purified by repeated precipitation and ethanol washing. The polymer gel was cut into pieces, soaked in anhydrous ethanol for 24 h, and washed five times with fresh ethanol, followed by vacuum drying at 40 °C for 48 h to remove unreacted monomers, residual OP-10 and small molecular impurities. A control sample, AM-AA, was synthesized using an identical polymerization procedure but without the addition of OP-10 or HDFDMA, and underwent the same ethanol washing and drying process to ensure consistent post-treatment conditions. The synthesis route for AM-AA-HDFDMA is illustrated in Figure 1.

#### 2.2.2. Structural Characterization of Hydrophobically Associating Salt-Tolerant Polymer

(1) Molecular Weight Determination: The intrinsic viscosities of AM-AA and AM-AA-HDFDMA were measured by the one-point method in accordance with the National Standard of the People’s Republic of China GB 12005.1-1989 [38] (Determination of Intrinsic Viscosity Number of Polyacrylamide). The viscosity-average molecular weights were subsequently calculated from the measured intrinsic viscosity values.

(2) Fourier-transform infrared spectroscopy (FT-IR): The dried AM-AA-HDFDMA samples were ground into fine powder and mixed with KBr at a mass ratio of 1:100 to prepare pellets by compression. The characteristic functional groups of the copolymers were characterized using an FT-IR spectrometer (Thermo Scientific, Waltham, MA, USA) over a scanning range of 4000–400^−1^ with a resolution of 4 cm^−1^ and 32 accumulated scans.

(3) Proton nuclear magnetic resonance (^1^H-NMR): Approximately 1–2 mg of the AM-AA-HDFDMA sample was placed in an NMR tube and dissolved in 0.55 mL of deuterium oxide (D_2_O) via ultrasonication. The ^1^H-NMR spectra were recorded on a 400 MHz spectrometer at room temperature, using D_2_O as the solvent and residual HDO as the internal standard to analyze the chemical environment and bonding configurations of the hydrogen atoms.

(4) Scanning electron microscopy (SEM): A polymer solution with a concentration of 1000 mg/L was prepared using deionized water. A single drop of the solution was uniformly applied onto conductive adhesive and freeze-dried in a vacuum freeze-dryer for 24 h. The dried specimens were then sputter-coated with gold to enhance conductivity. The micromorphology was observed using an SEM at magnifications of 500 times and 1000 times.

#### 2.2.3. Performance Evaluation of Polymer Solutions

(1) Viscosity Increasing Property: Polymer solutions with concentrations of 500–3000 mg/L were prepared with injection water and kept standing for 24 h at room temperature for full dissolution. The apparent viscosity of each solution was determined at 25 °C using a Brookfield DVT2 digital rotational viscometer (AMETEK Brookfield, Middleboro, MA, USA). The test shear rate was set at 7.34 s^−1^. The test temperature was precisely controlled by a circulating constant-temperature water bath with a temperature accuracy of ±0.1 °C. Each sample was tested in triplicate, and the average values were recorded to ensure reproducibility.

(2) Shear Resistance: A series of polymer solutions (500 to 3000 mg/L) were prepared following the procedure described in Section 1. The initial apparent viscosity (*η*_0_) was first measured. Subsequently, the polymer solutions were subjected to intense mechanical shearing using a high-speed blender at 30,000 r·min^−1^ for 30 s. After cooling the sheared solutions to 25 °C, the residual apparent viscosity (*η*_1_) was measured. The viscosity retention rate (*R*) was calculated according to Equation (1) to evaluate the shear resistance of the polymers:

(1) $$R=\frac{\eta_{1}}{\eta_{0}}\times 100\%$$

where *R* is the viscosity retention rate of the polymer solution (%); *η*_0_ and *η*_1_ are the apparent viscosities of the polymer solution before and after shearing (mPa·s), respectively.

(3) Thermal Stability: Polymer solutions of AM-AA-HDFDMA and AM-AA (2000 mg/L) were prepared using injection water as the solvent. The solutions were equilibrated in constant-temperature water baths at 30 °C, 40 °C, 50 °C, 60 °C, 70 °C, and 80 °C for 30 min, respectively. The apparent viscosity was measured at each temperature using a digital rotational viscometer to investigate the influence of temperature on the viscosity increasing property of the polymers.

(4) Salt Tolerance: AM-AA-HDFDMA and AM-AA solutions (2000 mg/L) were prepared using aqueous solutions of NaCl, CaCl_2_, and MgCl_2_ at varying concentrations as the solvents. All salt solutions were adjusted to pH 7.0 using dilute NaOH or HCl solutions before polymer dissolution. After standing at room temperature for 24 h to ensure complete hydration, the apparent viscosity of the polymer solutions in different electrolyte systems was determined at 25 °C. The effects of monovalent (Na^+^) and divalent (Ca^2+^, Mg^2+^) cations on the solution viscosity were systematically examined to evaluate the salt tolerance of the synthesized polymers.

(5) Resistance Factor (R_F_) and Residual Resistance Factor (R_RF_): The resistance coefficient and residual resistance coefficient of the polymer solution were determined by a sand-packed tube experiment. The test was performed at 65 °C, and the polymer solution was prepared with injection water. The sand-packed tube has an inner diameter of 2.5 cm and an effective length of 30 cm, and was filled with 40–80 mesh quartz sand. The specific experimental procedures are as follows: ① Inject injection water into the sand-packed tube at a rate of 1 mL/min, and record the stabilized injection pressure *P_1_* (water saturation stage); ② Inject 2500 mg/L polymer solution (prepared with injection water) into the sand-packed tube at 1 mL/min, and record the stabilized injection pressure P_2_ (polymer flooding stage); ③ Re-inject injection water into the sand-packed tube at 1 mL/min, and record the stabilized injection pressure P_3_ (subsequent water flooding stage).

(2) $$R_{F}=\frac{P_{2}}{P_{1}}$$

(3) $$R_{RF}=\frac{P_{3}}{P_{1}}$$

(6) Oil Displacement Performance Evaluation: Sand-pack flooding experiments were conducted to evaluate the enhanced oil recovery (EOR) performance of the polymers. The experiments were performed at 65 °C using degassed crude oil obtained from the oilfield (apparent viscosity of 43.8 mPa·s at 65 °C). The sand-pack model, with an inner diameter of 2.5 cm and an effective length of 30 cm, was uniformly packed with 40 to 80 mesh quartz sand, yielding an initial permeability of 1.247 D and a porosity of 32.01%. The specific experimental procedures were as follows: ① Water saturation: Formation water was injected into the sand-pack at a constant flow rate of 0.5 mL/min until the injection pressure stabilized, thereby completing the water saturation process. ② Oil saturation: Crude oil was injected at a flow rate of 0.1 mL/min until no water was produced at the outlet. The sand-pack was then sealed and aged for 24 h to complete the oil saturation process. ③ Initial water flooding: Injection water was injected at a rate of 2 mL/min until the water cut of the effluent reached 95%. The oil recovery during this initial water flooding stage (E_w_) was recorded. ④ Polymer flooding: A 2500 mg/L polymer solution (prepared with injection water) was injected at a flow rate of 2 mL/min with an injection volume of 0.4 pore volumes (PV). ⑤ Subsequent water flooding: Following the polymer injection, extended water flooding was performed using injection water at 2 mL/min until the water cut of the produced fluid reached 95% again. The total oil recovery (E_t_) for the entire displacement process was recorded.

Finally, the enhanced oil recovery contributed by the polymer flooding and the subsequent water flooding was calculated to quantitatively evaluate the oil displacement efficacy of the polymer.

## 3. Results and Discussion

### 3.1. Molecular Weight

The molecular weight measurement results are shown in Table 2. The intrinsic viscosity of AM-AA was 1802.6 cm^3^/g, and that of AM-AA-HDFDMA was 1849.6 cm^3^/g. The viscosity-average molecular weight of AM-AA was 9.419 × 10^6^ g/mol, and that of AM-AA-HDFDMA was 9.727 × 10^6^ g/mol.

### 3.2. FTIR

**Figure 2:**
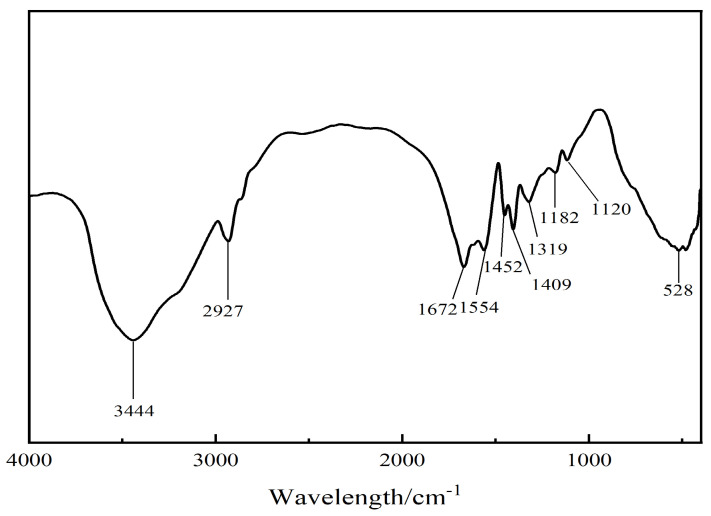
FT−IR spectrum of AM−AA−HDFDMA.

The FT-IR spectrum of AM-AA-HDFDMA is shown in Figure 2, and the assignments of the characteristic absorption bands are as follows: The broad peak at 3444 cm^−1^ is attributed to the N—H stretching vibration of the amide group (—CONH_2_) in the AM units, with the broadening effect resulting from intermolecular hydrogen bonding. The absorption at 2927 cm^−1^ corresponds to the C-H stretching vibrations of the alkyl groups (—CH_3_, —CH_2_) in the HDFDMA units. The peaks at 1672 cm^−1^ and 1409 cm^−1^ represent the C=O stretching vibration and the symmetric stretching vibration of the carboxyl groups (—COOH) in the AA units, respectively, while the band at 1554 cm^−1^ is assigned to the asymmetric stretching vibration of the —COOH group. The signals at 1452 cm^−1^ and 528 cm^−1^ are associated with the C—N stretching vibration and the NH_2_ out-of-plane wagging vibration of the amide groups, respectively. Regarding the HDFDMA units, the peak at 1319 cm^−1^ is attributed to the symmetric deformation vibration of the methyl group (—CH_3_), and the band at 1182 cm^−1^ corresponds to the C—O—C stretching vibration of the ester group (—COO—). Crucially, the characteristic absorption at 1120 cm^−1^ is assigned to the C-F stretching vibration of the fluorinated hydrophobic units, providing direct evidence for the successful incorporation of HDFDMA into the copolymer. The presence of all the aforementioned characteristic functional groups, coupled with the absence of significant residual monomer peaks, confirms the successful copolymerization of AA, AM, and HDFDMA, yielding the target product AM-AA-HDFDMA. Moreover, no characteristic benzene ring absorption peaks at 1610 cm^−1^, 1580 cm^−1^ and 1510 cm^−1^ assigned to OP-10 were detected, indicating that residual OP-10 was effectively removed after repeated ethanol purification.

### 3.3. ^1^H NMR

**Figure 3:**
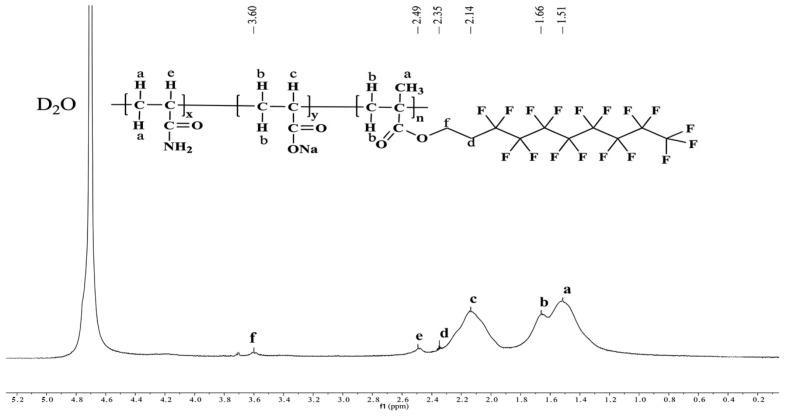
^1^H NMR spectrum of AM-AA-HDFDMA. a, backbone methylene protons of acrylamide units and α-methyl protons of perfluoroalkyl methacrylate units; b, backbone methylene protons of sodium acrylate and perfluoroalkyl methacrylate units; c, methine protons of sodium acrylate units; d, methylene protons adjacent to the perfluoroalkyl chain; e, methine protons of acrylamide units; f, methylene protons linked to the ester oxygen.

The ^1^H NMR spectrum of AM-AA-HDFDMA is presented in Figure 3. With D_2_O as the solvent, the assignments of the proton signals corresponding to each chemical shift (δ) are as follows: the peak at δ = 1.51 ppm (peak a) is attributed to the overlapping signals of the methyl protons (—CH_3_) in the HDFDMA unit and the methylene protons (—CH_2_) in the AM backbone. The resonance at δ = 1.66 ppm (peak b) corresponds to the overlap of the methylene protons (—CH_2_) in the AA backbone and the alkyl methylene protons (—CH_2_—) in the HDFDMA unit. The signal at δ = 2.14 ppm (peak c) is assigned to the methine protons connected to the side chains of the AA units. Peaks at δ = 2.35 ppm (peak d) and δ = 3.60 ppm (peak f) are ascribed to the methylene protons of —O—CH_2_—CH_2_— and the —O—CH_2_—CH_2_— group adjacent to the fluoroalkyl chain in the HDFDMA unit, respectively. The peak at δ = 2.49 ppm (peak e) represents the methine protons of the AM backbone.

Quantitative integration analysis was performed using the characteristic peaks at δ = 3.60 ppm (HDFDMA), δ = 2.14 ppm (AA) and δ = 2.49 ppm (AM). The calculated actual molar incorporation rate of HDFDMA in the copolymer was 0.31 mol%, which is highly consistent with the theoretical feeding ratio of 0.32 mol%. These results confirm the successful and quantitative copolymerization of the three monomers, which is in good agreement with the FT-IR results. In the range of δ = 6.5–7.5 ppm, no aromatic proton signals derived from the benzene ring structure of OP-10 were observed, further verifying that the residual OP-10 content is below the NMR detection limit.

### 3.4. Microstructure

**Figure 4:**
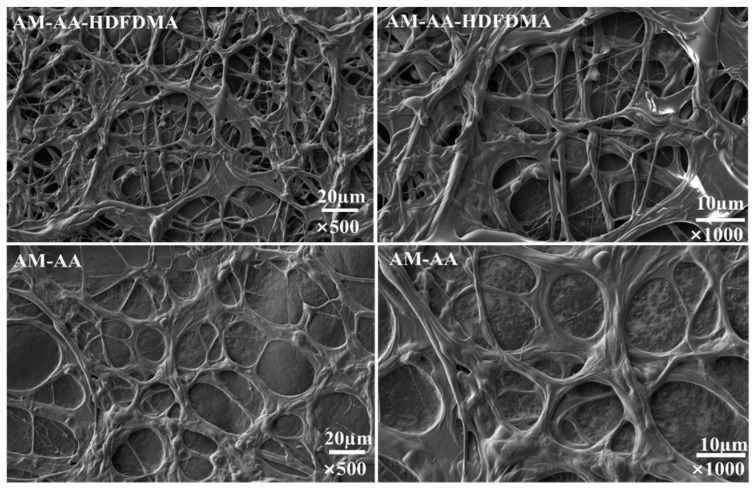
SEM images of the polymer solutions.

The SEM micro-morphologies of AM-AA and AM-AA-HDFDMA are displayed in Figure 4. It can be observed that AM-AA exhibits only a limited degree of molecular chain entanglement in aqueous solution. At a magnification of 1000×, its microstructure appears as a sparse mesh-like morphology, lacking a distinct three-dimensional (3D) spatial network. In contrast, AM-AA-HDFDMA presents a dense and continuous 3D spatial network structure, characterized by a significantly enhanced degree of chain entanglement. The observed morphological differences are mainly attributed to the introduction of fluorinated hydrophobic units. However, the introduction of fluorinated hydrophobic units into the AM-AA-HDFDMA chains enables hydrophobic association, which drives the aggregation of non-polar fluoroalkyl side chains. The microscopic morphology observed in SEM images is consistent with the predicted network structure formed by hydrophobically associating polymers in solution. Such a structure significantly reinforces the structural strength of the polymer solution, providing a microscopic foundation for its superior shear resistance and salt tolerance.

### 3.5. Viscosity Increasing Property

**Figure 5:**
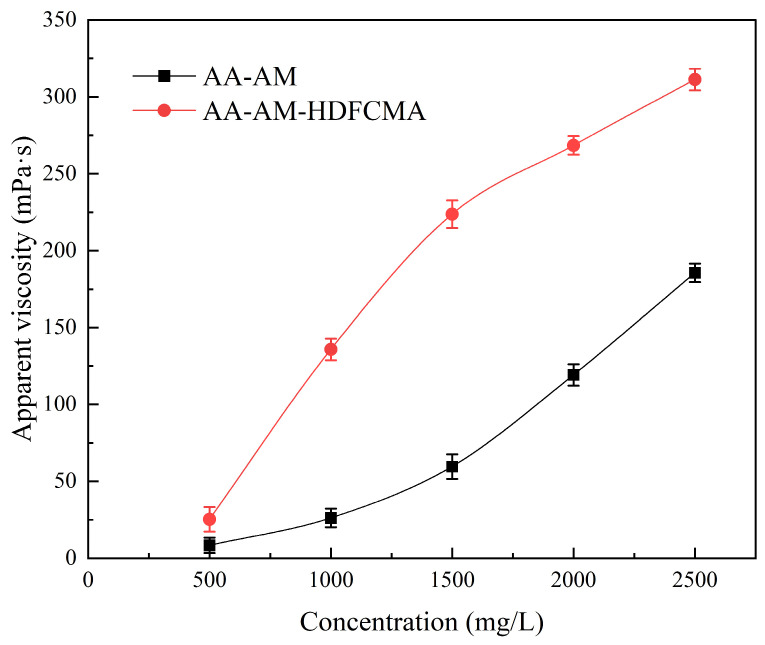
Viscosity increasing property of the polymers.

The dependence of the apparent viscosity of AM-AA and AM-AA-HDFDMA solutions on mass concentration is illustrated in Figure 5. Although the apparent viscosity of both polymer solutions increases with rising concentration, their thickening rates differ significantly. The viscosity of the AM-AA solution increases slowly, exhibiting a relatively poor thickening performance. In contrast, while the viscosity of AM-AA-HDFDMA follows a similar trend to AM-AA below 1000 mg/L, it undergoes an exponential increase once the concentration exceeds this threshold, estimated as the critical association concentration (CAC) from the viscosity inflection point. At 25 °C, the apparent viscosity of the 2000 mg/L AM-AA-HDFDMA solution reaches 268.41 mPa·s, which is 2.25 times higher than that of AM-AA (119.10 mPa·s) at the same concentration.

This phenomenon can be explained by the transition in molecular interactions. Below the CAC, AM-AA-HDFDMA chains exist primarily as individual coils where intramolecular hydrophobic associations dominate, contributing minimally to the viscosity. Above the CAC, a transition from intramolecular to intermolecular hydrophobic association occurs. The hydrophobic aggregation of fluoroalkyl side chains promotes extensive chain entanglement and the formation of a robust 3D spatial network, which significantly enhances the internal frictional resistance and leads to a sharp increase in apparent viscosity. Conversely, AM-AA lacks hydrophobic units and relies solely on chain entanglement and hydrogen bonding, resulting in a much lower thickening efficiency compared to AM-AA-HDFDMA.

### 3.6. Shear Resistance

**Figure 6:**
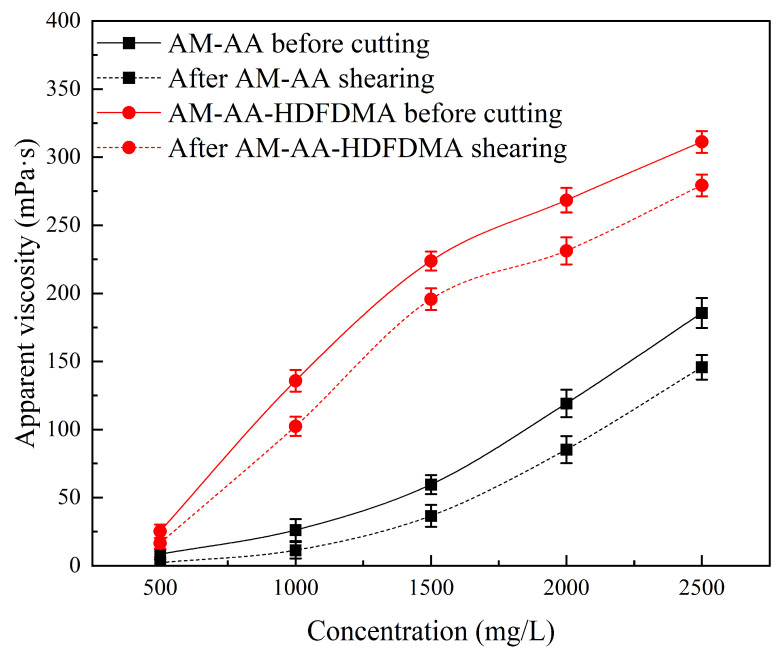
Shear resistance of the polymer solutions.

Figure 6 illustrates the changes in the apparent viscosity of AM-AA and AM-AA-HDFDMA solutions before and after mechanical shearing. Following exposure to high-speed shearing at 30,000 r·min^−1^ for 30 s, both polymer solutions exhibit varying degrees of viscosity reduction. Notably, the viscosity loss of the AM-AA-HDFDMA solution is significantly lower than that of AM-AA, demonstrating a much higher viscosity retention rate. At a concentration of 2000 mg/L, the viscosity retention rate of AM-AA is only 71.56%, whereas that of AM-AA-HDFDMA reaches 88.91% (an increase of 17.35 percentage points). At 2500 mg/L, the retention rate of AM-AA-HDFDMA is 11.27% higher than its counterpart, indicating superior shear resistance.

The underlying mechanism for this disparity lies in their molecular architectures. Linear AM-AA molecular chains are prone to disentanglement and possible mechanical degradation under high shear, resulting in obvious viscosity reduction. In contrast, the 3D spatial network of AM-AA-HDFDMA is constructed via hydrophobic associations. These non-covalent interactions possess dynamic reversibility: while high shear forces may temporarily disrupt some intermolecular associations and loosen the network, these hydrophobic domains can re-associate once the shear stress is removed. This rapid recovery of the 3D network architecture allows the solution viscosity to rebound, resulting in a substantially improved viscosity retention rate.

### 3.7. Thermal Resistance

**Figure 7:**
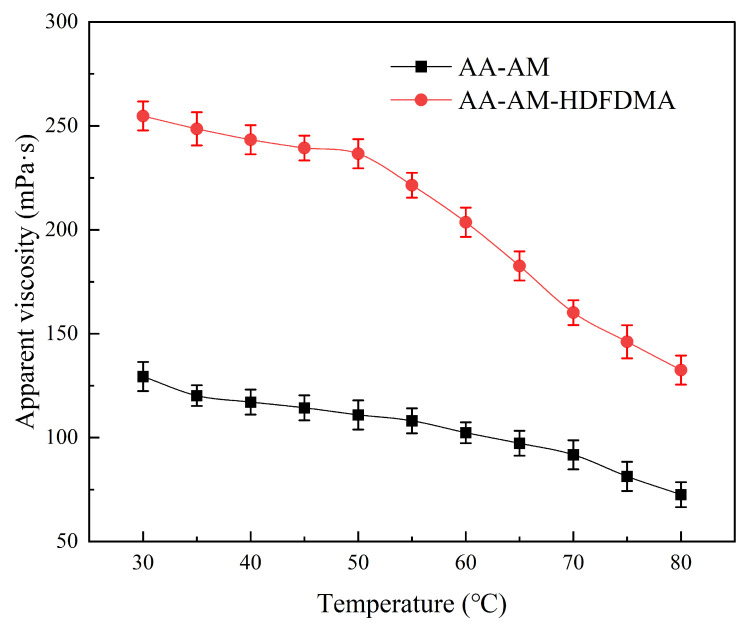
Temperature resistance of the polymers.

The variation of apparent viscosity with temperature for AM-AA and AM-AA-HDFDMA solutions is illustrated in Figure 7. As the temperature rises from 30 °C to 80 °C, a gradual decline in apparent viscosity is observed for both polymer solutions. This trend is attributed to the intensified thermal motion of water molecules at elevated temperatures, which thins the hydration layer of the polymer chains and increases chain flexibility, thereby reducing both the degree of entanglement and the internal frictional resistance. Notably, at any given temperature, the apparent viscosity of AM-AA-HDFDMA remains significantly higher than that of AM-AA, exhibiting a slower decline rate and superior thermal stability.

Specifically, the apparent viscosity of the 2000 mg/L AM-AA-HDFDMA solution is 254.76 mPa·s at 30 °C and decreases to 132.46 mPa·s at 80 °C, representing a viscosity retention rate of 51.99%. In contrast, the AM-AA solution shows a drop from 129.36 mPa·s to 72.19 mPa·s over the same range, with a retention rate of 55.81%. Notably, although the normalized viscosity retention rate of AM-AA-HDFDMA is slightly lower than that of AM-AA, the absolute viscosity of AM-AA-HDFDMA remains significantly higher than that of AM-AA at all tested temperatures. For EOR applications, the absolute viscosity of the polymer solution under reservoir conditions is the more critical parameter determining displacement efficiency. The better temperature tolerance of AM-AA-HDFDMA is mainly attributed to the strong hydrophobic association and high chemical stability of fluorinated segments. At high temperatures, the hydrophobic association can partially offset the adverse effects of chain disentanglement, maintaining the stability of the 3D spatial network and mitigating viscosity loss.

### 3.8. Salt Resistance

**Figure 8:**
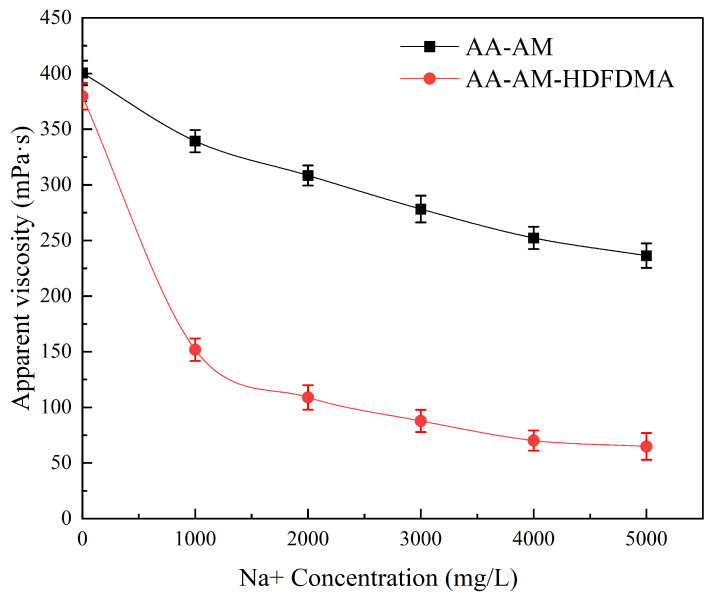
Na^+^ resistance of the polymer solutions.

**Figure 9:**
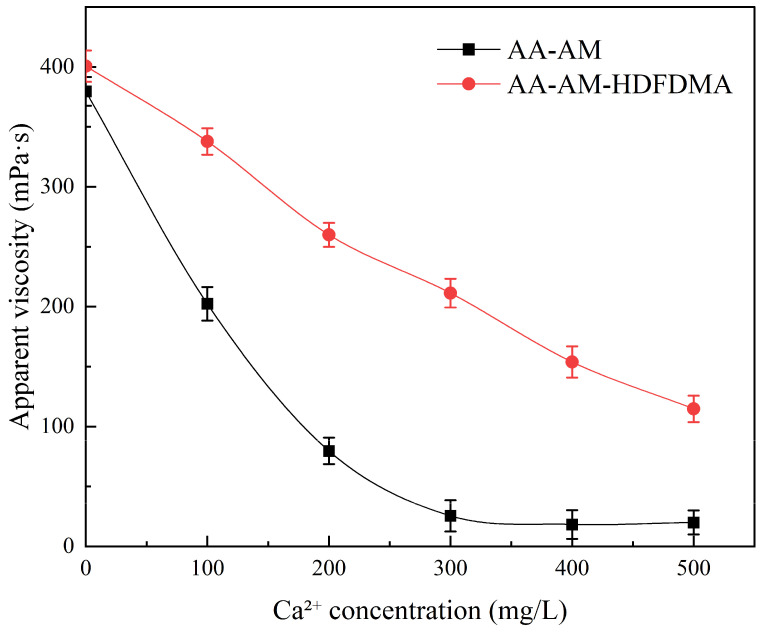
Ca^2+^ resistance of the polymer solutions.

**Figure 10:**
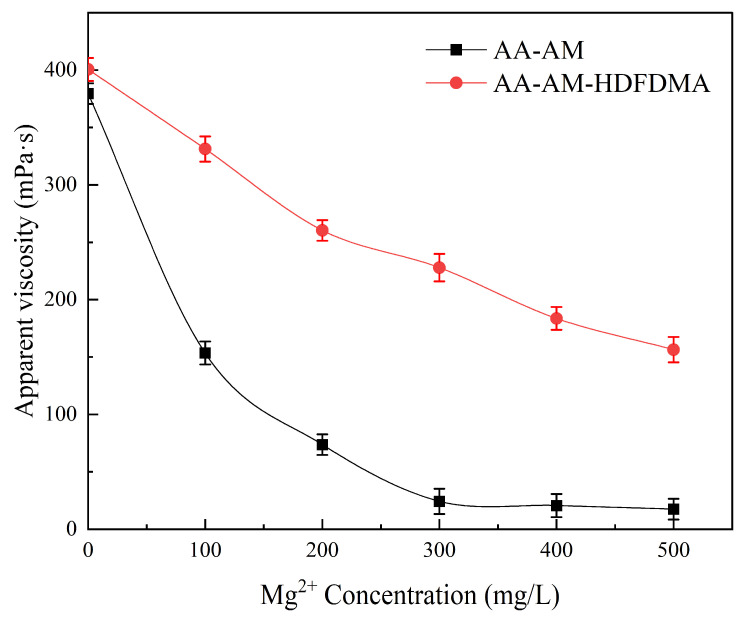
Mg^2+^ resistance of the polymer solutions.

The presence of Na^+^, Ca^2+^, and Mg^2+^ in oilfield formation water constitutes the primary cause of viscosity loss in polymer solutions, with multivalent metal ions (Ca^2+^ and Mg^2+^) exerting a more pronounced impact. Figure 8, Figure 9 and Figure 10 illustrate the effects of these ions on the apparent viscosity of AM-AA-HDFDMA and AM-AA. While the viscosity of both polymers declines with increasing salt concentration, the reduction rate for AM-AA is significantly higher, leading to a near-total loss of its thickening ability at high salinities. Conversely, AM-AA-HDFDMA maintains a robust apparent viscosity under high-salt conditions, demonstrating exceptional salt tolerance, particularly toward multivalent ions. In a 5000 mg/L Na^+^ solution, the viscosity of 2000 mg/L AM-AA-HDFDMA is 308.40 mPa·s, which is 199.47 mPa·s higher than that of AM-AA. Remarkably, in 600 mg/L Ca^2+^ or Mg^2+^ solutions, AM-AA-HDFDMA retains substantial viscosity, while the viscosity of AM-AA approaches zero. The underlying mechanisms for these observations are distinct. In conventional AM-AA, the carboxyl groups (—COO^−^) readily undergo electrostatic complexation with metal ions, which screens the electrostatic repulsion between molecular chains, leading to severe salting-out and chain coiling.

In contrast, the fluorinated hydrophobic units in AM-AA-HDFDMA construct a dense and stable 3D spatial network via strong hydrophobic association, which is consistent with the SEM characterization results. This network architecture can effectively resist the interference of metal ions on polymer chains and mitigate the chain collapse caused by electrostatic shielding, which is believed to be the main reason for its superior salt tolerance.

Based on the significant difference in salt sensitivity between AM-AA and AM-AA-HDFDMA under the same carboxyl content, we reasonably infer that the strong hydrophobic and oleophobic fluorocarbon chains may form a hydrophobic microdomain shield on the surface of the polymer molecular chains. This shield may reduce the contact probability between high-valence metal ions and carboxyl groups on the polymer backbone, thus further alleviating the salting-out effect caused by the complexation of carboxyl groups and metal ions. Direct verification of this interfacial behavior will be systematically investigated in our follow-up work.

### 3.9. R_F_ and R_RF_

The results of *R_F_* and *R_RF_* are listed in Table 3. For the 2500 mg/L AM-AA solution and AM-AA-HDFDMA solution, the corresponding resistance coefficients are 12.4884 and 19.6235, respectively. This indicates that the AM-AA-HDFDMA solution has a superior ability to improve the water–oil mobility ratio and can build higher seepage resistance in porous media. The residual resistance coefficients of the two solutions are 2.0698 and 2.9529 correspondingly. It reveals that AM-AA-HDFDMA exhibits a stronger capacity to reduce water-phase permeability. After the injection of AM-AA-HDFDMA solution, the water flooding pressure increases, which is beneficial to expanding the swept volume of injected water.

### 3.10. Oil Displacement Performance Evaluation

**Figure 11:**
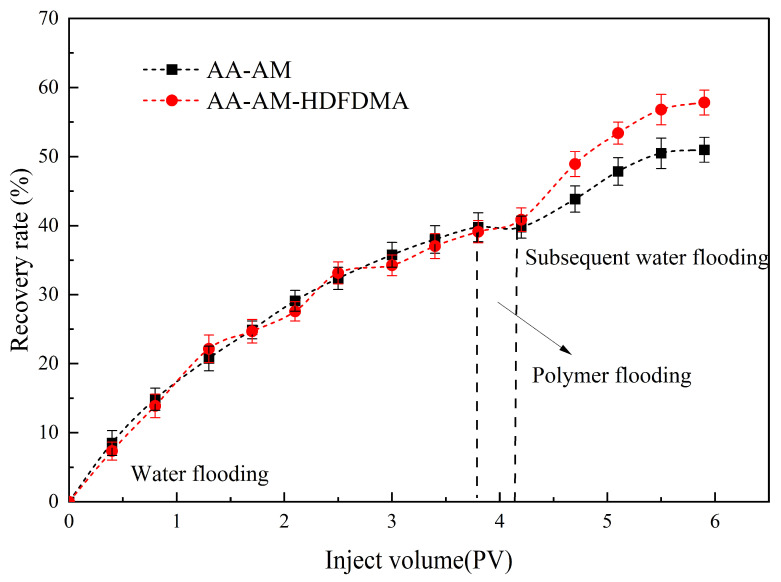
Enhanced oil recovery (EOR) as a function of injected pore volume (PV).

The oil recovery performance of AM-AA and AM-AA-HDFDMA solutions is presented in Figure 11. At 65 °C, following the injection of 0.4 PV (pore volume) of a 2500 mg/L polymer solution, the AM-AA solution achieved an enhanced oil recovery (EOR) of 11.21%. In contrast, the AM-AA-HDFDMA solution reached an EOR of 16.97%, representing a 5.76 percentage point increase over AM-AA and demonstrating superior oil displacement efficiency.

The effectiveness of a polymer in oil displacement primarily depends on its thickening capacity and environmental adaptability. AM-AA-HDFDMA shows excellent thickening performance, which can increase injected water viscosity, optimize water–oil mobility ratio and enlarge oil sweep volume. Furthermore, its robust resistance to temperature, salinity, and shear allows it to maintain a high viscosity in harsh reservoir environments (high temperature and high salinity), preventing the degradation of displacement efficiency caused by viscosity loss during injection. Additionally, the 3D spatial network of AM-AA-HDFDMA effectively plugs high-permeability layers, diverting the injected water toward low-permeability zones and enhancing the microscopic displacement efficiency. Conversely, AM-AA suffers from severe viscosity loss at 65 °C, making it difficult to effectively improve the mobility ratio, which leads to inferior oil recovery results.

It should be noted that the oil displacement comparison was performed at the same polymer concentration (2500 mg/L) rather than at matched viscosity. Therefore, the higher EOR efficiency of AM-AA-HDFDMA may be partially attributed to its higher solution viscosity. However, in practical field applications, polymer solutions are typically prepared and injected at a fixed mass concentration, and the higher viscosity of AM-AA-HDFDMA at the same concentration represents a significant practical advantage.

## 4. Practical Applicability and Potential Limitations

The AM-AA-HDFDMA polymer synthesized in this work has good application potential for enhanced oil recovery in high-temperature and high-salinity reservoirs. For industrialization, the polymer is prepared via aqueous solution polymerization with mild reaction conditions (40°C, atmospheric pressure), simple operation and no complex post-treatment, which is fully compatible with the existing industrial production process of commercial oilfield polyacrylamide, and has excellent scale-up feasibility. In terms of economy, the dosage of fluorinated monomer HDFDMA is only ~0.8 wt% of the total monomers, which will not significantly increase the synthesis cost; meanwhile, the ultra-low critical association concentration (1000 mg/L) makes its effective field dosage much lower than conventional commercial HPAM, which can effectively reduce the application cost of chemical flooding.

For potential limitations, the environmental risk of fluorinated monomers is the core concern. In this work, the extremely low addition of fluorinated monomer and the expected high adsorption retention of the high-molecular-weight polymer in reservoir porous media are expected to reduce the migration risk of fluorine-containing components with produced water. Meanwhile, the development of high-performance short fluorocarbon chain alternatives with lower environmental risk will be the core direction of our follow-up research.

## 5. Conclusions

(1) A fluorinated hydrophobically associating polymer AM-AA-HDFDMA was successfully synthesized via aqueous solution copolymerization using AM, AA as comonomers and HDFDMA as fluorinated hydrophobic monomer, with APS-NaHSO_3_ as redox initiation system. FT-IR and ^1^H-NMR results confirmed the successful copolymerization of three monomers. SEM observations indicated that the introduction of HDFDMA contributes to the formation of dense continuous 3D network microstructure in aqueous solution. Within the experimental scope of this work, the suitable polymerization conditions were determined as: total monomer mass fraction 25%, initiator dosage 0.3 wt% of total monomers, reaction temperature 40 °C and reaction time 4.5 h.

(2) Compared with the binary AM-AA copolymer, AM-AA-HDFDMA presents better thickening ability. At 25 °C, the apparent viscosity of 2000 mg/L AM-AA-HDFDMA reaches 268.41 mPa·s, about 2.25 times that of AM-AA under the same condition. The concentration of 1000 mg/L is identified as the critical association concentration (CAC) according to the inflection point of viscosity–concentration curve. The sharp viscosity increase above 1000 mg/L implies the enhancement of intermolecular hydrophobic association among fluorinated segments.

(3) Under the tested experimental conditions, AM-AA-HDFDMA shows better shear resistance, thermal stability and salt tolerance than the AM-AA counterpart. After high-speed shearing at 30,000 r·min^−1^ for 30 s, its viscosity retention is higher than that of AM-AA. The polymer maintains stable viscosity as temperature increases to 80 °C, and also retains high apparent viscosity in high-concentration NaCl, CaCl_2_ and MgCl_2_ solutions. The superior comprehensive performance of AM-AA-HDFDMA is mainly attributed to the hydrophobic association network formed by fluorinated HDFDMA units.

(4) Sand-pack flooding tests show that the injection of 0.4 PV 2500 mg/L AM-AA-HDFDMA solution at 65 °C increases oil recovery by 16.97%, which is 5.76 percentage points higher than that of AM-AA. The results indicate that the synthesized fluorinated polymer has good application potential as an EOR agent for high-temperature and high-salinity reservoir conditions. Further systematic research on molecular-level mechanism and field adaptability will be carried out in our follow-up work.

## Figures and Tables

**Table 1:** Ion components of simulated formation water.

Ion Species	Na^+^	K^+^	Ca^2+^	Mg^2+^	HCO_3_^−^	SO_4_^2−^	Cl^−^
Ion content of injected water (mg/L)	1030	29	58	29	199	58	1642
Ion content of formation water (mg/L)	2740	131	1924	133	95	209	10,248

**Table 2:** Intrinsic viscosity and viscosity-average molecular weight of AM-AA and AM-AA-HDFDMA.

Solution	*t*_1_ (s)	*t*_2_ (s)	*t*_3_ (s)	Average Flow Time (s)	Intrinsic Viscosity (cm^3^/g)	Viscosity-Average Molecular Weight
1 mol/L NaCl	117.8	118.1	117.9	117.9	/	/
800 mg/L AM-AA	392.2	392.5	391.9	392.2	1802.6	9,419,106.3
800 mg/L AM-AA-HDFDMA	403.8	402.1	401.6	402.5	1849.6	9,727,139.7

**Table 3:** Experimental results of R_F_ and R_RF_.

Polymer Solution	P_1_ (MPa)	P_2_ (MPa)	P_3_ (MPa)	R_F_	R_RF_
AM-AA solution	0.0086	0.1074	0.0178	12.4884	2.0698
AM-AA-HDFDMA solution	0.0085	0.1668	0.0251	19.6235	2.9529

Note: These are single-run experimental results. Repeated experiments will be conducted in follow-up work to verify data reproducibility.

## Data Availability

The original contributions presented in this study are included in the article. Further inquiries can be directed to the corresponding author.
